# Development of a Novel Modular Footwear Setup for Testing the Isolated Biomechanical Effects of Footwear Features

**DOI:** 10.1002/jfa2.70203

**Published:** 2026-08-21

**Authors:** Hadi Sarlak, Kamran Shakir, Giulia Rogati, Giorgia Sartorato, Alberto Leardini, Lisa Berti, Paolo Caravaggi

**Affiliations:** ^1^ Department of Biomedical and Neuromotor Sciences Alma Mater Studiorum—Università di Bologna Bologna Italy; ^2^ Movement Analysis Laboratory and Functional Evaluation of Prostheses IRCCS Istituto Ortopedico Rizzoli Bologna Italy; ^3^ Independent Researcher Treviso Italy; ^4^ Physical Medicine and Rehabilitation Unit IRCCS Istituto Ortopedico Rizzoli Bologna Italy

**Keywords:** in‐shoe pressure, lower‐limb kinematics, midsole, modular footwear, plantar pressure, repeatability, therapeutic shoes

## Abstract

**Background:**

In footwear science, the effect of specific design features on biomechanical parameters during common motor tasks is often confounded by simultaneous changes in other shoe conditions. This study proposes and assesses the repeatability of a new tool, namely the Modular Footwear Setup (MFS), to assess the effects of midsole modifications on lower‐limb joint kinematics and in‐shoe pressure measurements.

**Methods:**

The MFS uses a micro‐hook‐and‐loop fastening system and a custom alignment device to enable fast and reliable midsole attachment/detachment to/from the upper. Repeatability of joint kinematics and in‐shoe pressure parameters was tested in 10 healthy participants (5M, 5F; age = 33.2 ± 9.2 yrs; BMI = 21.5 ± 2.8 kg*m^−2^) across three walking sessions. The effect of the MFS fixation method was assessed by comparing relevant biomechanical outcomes to those of a control shoe featuring the same upper and midsole. Comfort‐related outcomes were reported using a visual analog scale.

**Results:**

Statistical Parametric Mapping analysis did not identify significant differences in joint kinematics between conditions. No significant differences were observed in pressure parameters at any foot region between MFS and control, except for the peak pressure at the rearfoot. The MFS demonstrated good‐to‐excellent inter‐session repeatability (ICC 0.84–0.97) for mean and peak pressure. Participants reported similar levels of comfort and stability in both shoes.

**Conclusion:**

The findings of this study suggest that the MFS has the potential to be a reliable tool for evaluating the effects of midsole features on relevant biomechanical parameters. This modular approach may improve data‐driven footwear design by improving the consistency of biomechanical measurements.

## Graphical abstract

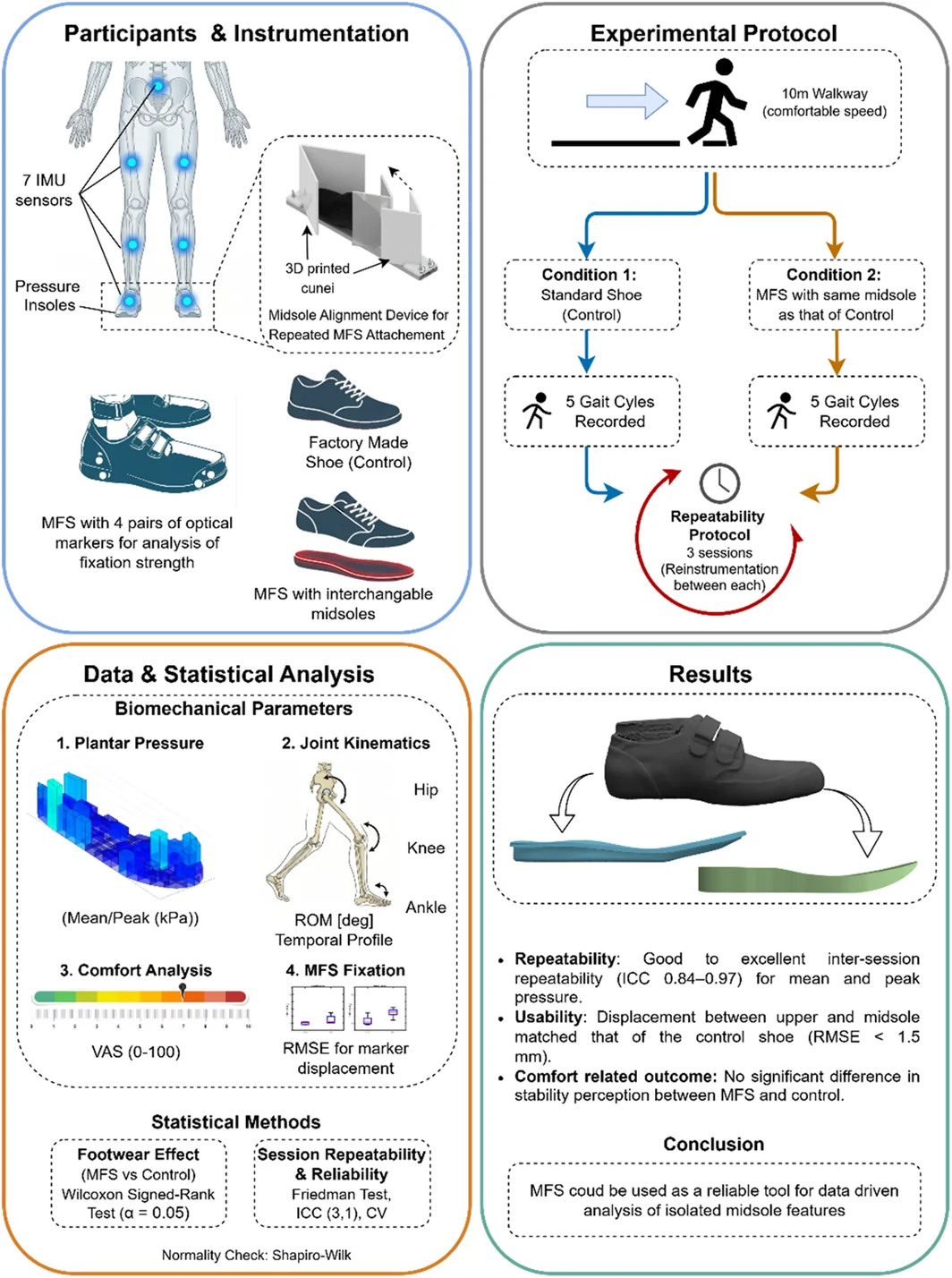

## 1 Introduction

Footwear is a protective interface between the foot and the ground and a mechanical intervention that modifies how loads are transmitted through the lower limb during tasks of daily living. In clinical and therapeutic scenarios, understanding this mechanical interaction is critical since design features such as sole stiffness, rocker geometry, outsole shape, and material properties may significantly affect plantar pressure distribution, rollover mechanics, and joint kinematics [1, 2, 3, 4]. These biomechanical outcomes are particularly relevant to the design of offloading solutions for people with diabetes at risk of ulceration [5, 6]. Plantar offloading is usually addressed by specific therapeutic, bespoke or off‐the‐shelf shoes, which are key components of ulcer prevention and management [7]. It has been shown that specific footwear design features, such as rockers and rigid midsole/outsole materials, help redistribute plantar pressure and lower reulceration rates [8, 9].

Evaluating the isolated effects of individual footwear features is crucial for clinical research and helps promote a shift from experience‐based methods to a more data‐driven approach in footwear manufacturing [10]. However, footwear‐related studies often involve multiple design modifications simultaneously, such as variations in fit due to changing interventions, upper construction, last shape, and insole characteristics, thus making it difficult to determine cause‐and‐effect relationships between specific footwear features and observed biomechanical outcomes [1]. This challenge is compounded by variability in study protocols and in the properties of intervention and control shoes, which can lead to biased or incomplete results and severely limit the strength of footwear design recommendations [11, 12].

An approach to evaluating the effects of isolated footwear features on biomechanical parameters is to use modular or modifiable designs that allow the modification of a single feature while keeping the rest of the shoe unchanged. Cavanagh et al. (1997) developed an experimental modular extra‐depth shoe that used the same upper and footbed while allowing different rocker‐bottom modules to be tested in terms of plantar pressure distribution [13]. More recently, a modular shoe with interchangeable heel components that can be attached to the same upper using screws has been proposed to test the effect of heel rocker parameters on plantar pressure distribution [14]. Another approach to test the isolated effects of midsole geometry used an experimental shoe with rails and sliders for continuous forefoot rocker adjustment [15]. A custom 3D‐printed rocker midsole with varying rocker radii and longitudinal bending stiffness has also been reported [4]. The forefoot bending motion of the midsole was restricted with cable ties, ensuring that the observed pressure changes were attributable to outsole parameters rather than to differences in the upper or fit. To the best of the authors' knowledge, none of the reported experimental shoes have been thoroughly assessed in terms of the repeatability of biomechanical measurements.

To overcome these limitations, the present study had two main aims: (1) to develop a modular footwear setup (MFS) to test the isolated effects of different midsole interventions on biomechanical parameters, such as foot and lower‐limb kinematics and plantar pressure distribution, and (2) to assess the repeatability of relevant biomechanical measurements during walking while wearing the MFS.

## 2 Materials and Methods

### 2.1 Population

Ten healthy adults (5 M, 5 F; age = 33.2 ± 9.2 yrs; height = 1.73 ± 0.11 m; weight = 65.0 ± 14.1 kg; BMI = 21.5 ± 2.8 kg*m^−2^) were enrolled in the study. The inclusion criteria were age between 18 and 65 years, having a shoe size of 43 or 37 EU, and the ability to provide informed consent and comply with the tasks. Exclusion criteria comprised having lower‐limb musculoskeletal issues or surgeries, diabetes, peripheral neuropathy, and foot deformities.

The study received approval from the local ethics committee (AVEC #647/2025/Sper/IOR). All participants were informed about the data collection process and signed informed consent forms to participate.

### 2.2 The Modular Footwear Setup

**FIGURE 1:**
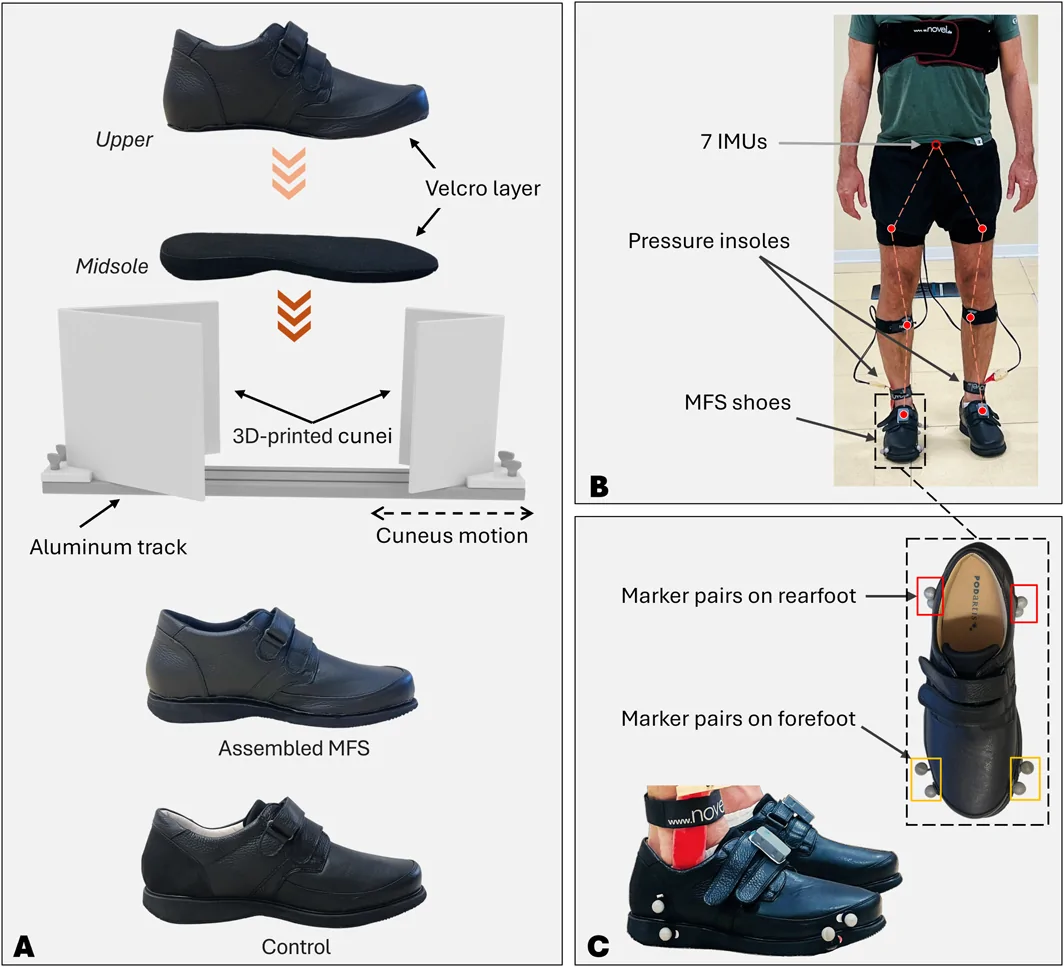
Brief graphical description of the experimental protocol. (A) Operation of the alignment device: the midsole is kept firmly in place by two sliding cunei that can be adjusted and fixed along the aluminum track using screws. The participant wearing the upper gently slides the foot downwards, guided by the cunei, onto the midsole. (B) An exemplary participant instrumented with in‐shoe pressure insoles and seven IMUs (the IMU on the pelvis is attached posteriorly to the top of the sacrum, approximately on S1). (C) Pairs of reflective markers attached to the shoes were used to measure the relative movement between the upper and midsole during walking.

The MFS was designed at the authors' institution to test the effect of midsole/outsole materials and shapes on biomechanical parameters. The MFS comprises three main components: a comfortable upper from an off‐the‐shelf orthopedic shoe (Botero, Podartis, Italy) with a soft insole‐board (the part on the bottom side of the shoe upper to provide a surface for attaching the midsole); a collection of interchangeable midsoles; and a micro‐hook‐and‐loop fastener layer (Velcro, US) to allow easy fixation of the midsoles to the shoe upper (Figure 1A). A custom alignment device was designed for accurate and repeatable midsole attachment to the upper (Figure 1A). The device comprises an 8 cm wide aluminum track fitted with two 3D‐printed (Stratasys F370, US) cunei (20 cm high, 60 deg angle) made from Acrylonitrile Butadiene Styrene (ABS) filament. One of the two cunei can slide and be locked at any point along the aluminum track, ensuring firm positioning of the midsole for attachment. The upper, worn by the participant, slides in and is guided along the cunei until it attaches to the midsole.

### 2.3 Experimental Protocol

Participants were asked to walk at a comfortable walking speed along a 10 m walkway in two shod conditions: (1) wearing a standard comfortable orthopedic footwear (Botero, Podartis, Italy), and (2) wearing the MFS featuring an identical midsole. The order was randomized for each participant, and walking speed was controlled using a stopwatch to be within 10% of the first walking trial. Five participants wore EU size 43, and five wore EU size 37. They were asked to perform common tasks of daily living (e.g., walking, ascending/descending stairs, and tiptoe standing) to familiarize themselves with the shoes before data acquisition. Each participant was instrumented with seven Inertial Measurement Units (IMUs; Ultium Motion, Noraxon, US), sampling at 200 Hz. Four IMUs were attached to the shanks and thighs of the left and right legs using Velcro straps. One IMU was secured to the sacrum (approximately on S1), and two were attached to the left and right shoes using double‐sided adhesive tape (Figure 1B). IMUs were placed on the body segments and calibrated according to the manufacturer's recommendations before each data acquisition. Temporal profiles of hip, knee, and ankle joint rotations in the sagittal and frontal planes were collected and normalized over gait cycle duration. Range of motion (ROM) was calculated for each joint as the absolute difference between the maximum and minimum angles in gait. Each shoe was fitted with a pressure insole featuring 99‐capacitive sensors (Pedar X, Novel, Germany) sampling at 100 Hz (Figure 1B). The following pressure parameters were recorded for each participant in each condition: mean and peak plantar pressure [kPa] at the whole foot, rearfoot, midfoot, and forefoot regions of interest (ROIs). Data were analyzed using a custom software based on MATLAB (MathWorks, US). For each participant, biomechanical parameters were averaged over 5 gait cycles in each condition.

In addition, each participant completed a Visual Analog Scale (VAS) questionnaire to assess the overall perceived comfort, walking stability, and comfort at push‐off for each shoe condition.

### 2.4 Statistical Analysis

Plantar pressure data were checked for normality using the Shapiro–Wilk test. Due to the small sample size (*n* = 10) and the nonnormal distribution detected in several parameters, paired Wilcoxon Signed‐Rank Tests were used to assess possible differences in biomechanical parameters between the MFS and control shoes. A Bonferroni correction was applied to the significance level to account for multiple paired comparisons (*α* = 0.0125) of plantar pressure assessment. Statistical Parametric Mapping (SPM) [16] was used to assess differences in the temporal profiles of joint rotations between the two shoe conditions. The VAS data were analyzed using the Wilcoxon Signed‐Rank test.

### 2.5 Repeatability Analysis

To assess the repeatability of biomechanical measurements when wearing the MFS, pressure and kinematic data were recorded for each participant across three sessions. At the end of each session, the IMUs were detached from the body segments, the pressure insoles were removed from the uppers, and the midsole components were detached from the uppers. Before each new session, the participant was reinstrumented with the IMUs, and the pressure insoles were inserted in the MFS uppers. The midsoles were reattached to the upper via the alignment device. Repeatability of pressure parameters across sessions was quantified using the Intraclass Correlation Coefficient (ICC 3, 1), the Standard Error of Measurement (SEM), and the Coefficient of Variation (CV). Repeatability of joint ROM measurements was assessed via ICC [2, 17] and CV. All analyses were performed using MATLAB (R2025a, MathWorks, US).

### 2.6 Analysis of the Midsole–Upper Fixation Strength

To assess the stability of the fixation between the MFS midsole and upper, four pairs of spherical reflective markers were attached to the midsole and upper at the rearfoot and forefoot (Figure 1C). Markers' trajectories were tracked via an 8‐camera motion analysis system (Vicon Motion Systems Ltd, Oxford, UK) sampling at 100 Hz. The temporal profile of the relative distance between two markers placed on the midsole and upper of each pair was calculated over three gait cycles in a subsample of five participants in both MFS and control shoe conditions. For each pair of markers, the Root Mean Square Error (RMSE) of the distance [mm] was calculated with respect to that in the static position.

## 3 Results

This study evaluated the performance of the MFS across several domains: biomechanical assessment of the effect of the fixation system on joint kinematics and in‐shoe pressure; inter‐session repeatability of biomechanical outcomes; mechanical fixation stability; and user experience. No difference in walking speed (m*s^−1^) was observed between the control and MFS conditions (median speed [IQR, 25th–75th percentile]: control = 1.27 [1.12–1.36], MFS = 1.26 [1.15–1.34]; Wilcoxon *p* = 0.85). Similarly, no difference was observed in walking speed between the three walking sessions of the repeatability analysis (median speed [IQR, 25th–75th percentile]: session 1 = 1.26 [1.15–1.34], session 2 = 1.28 [1.19–1.38], session 3 = 1.30 [1.22–1.44]; Friedman *p* = 0.19).

**FIGURE 2:**
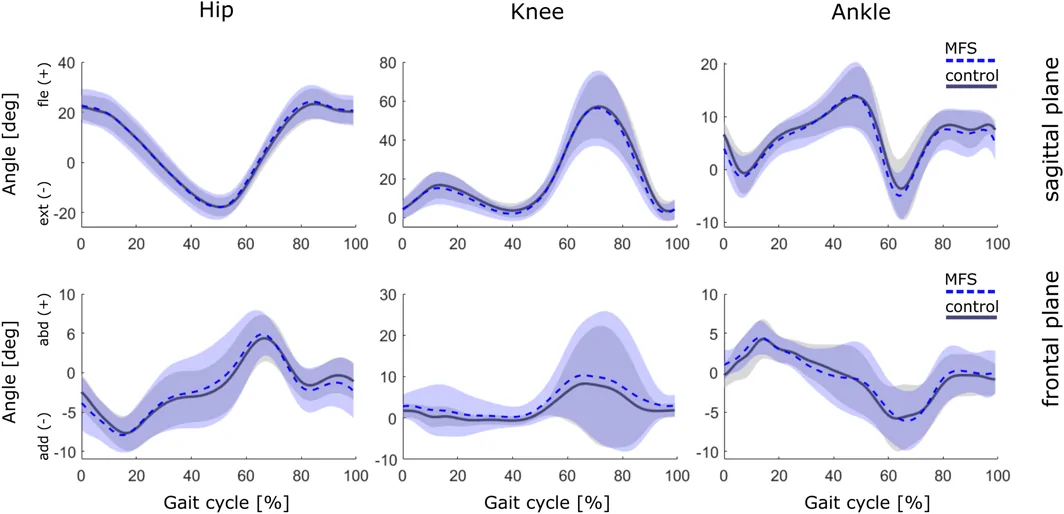
Temporal profiles (± 1SD) of hip, knee, and ankle joint rotations in the sagittal (top) and frontal (bottom) plane over time‐normalized gait cycle in the MFS and in the control shoe.

Figure 2 reports the hip, knee, and ankle joint rotations in the sagittal and frontal planes over the time‐normalized gait cycle in the MFS and control shoe conditions. SPM analysis did not show statistically significant differences in temporal profiles across any time frame (see Supporting Information S1: Appendix A for *t*‐plots).

The inter‐subject distribution of pressure parameters in all ROIs is summarized in Table 1. No statistically significant differences in pressure parameters were observed across ROIs between shoe conditions, except for rearfoot peak pressure, which was slightly lower in the control shoe.

**TABLE 1:** Inter‐participant mean and peak pressure distributions in the MFS and in the control shoe in four plantar ROIs.

	Region	MFS median [25%, 75%]	Control median [25%, 75%]	Wilcoxon *p*‐value
Mean pressure (kPa)	Whole foot	172 [149,187]	159 [145, 186]	0.575
	Rearfoot	101 [98, 117]	92 [90, 104]	0.028
	Midfoot	49 [34, 54]	46 [32, 53]	0.203
	Forefoot	107 [95, 133]	107 [94, 141]	0.799
Peak pressure (kPa)	Whole foot	295 [258, 335]	284 [241, 402]	0.959
	Rearfoot	244 [224, 305]	225 [209, 273]	0.005*
	Midfoot	72 [53, 83]	75 [56, 82]	0.074
	Forefoot	269 [248, 334]	271 [237, 402]	0.646

* indicate statistically significant differences between shoe conditions (*α* = 0.012).

Pressure measurements demonstrated high repeatability across the three sessions in most ROIs (Table 2). Specifically, mean and peak pressure at the midfoot and forefoot showed excellent repeatability (ICC > 0.9). Mean pressure at the rearfoot showed good repeatability (ICC > 0.8). Similarly, the ankle range of motion showed good repeatability (ICC > 0.8) across sessions (Table 3), but slightly lower ICC for hip and knee joints. CVs were lower than 8% across all joints.

**TABLE 2:** Repeatability of pressure measurements across three sessions wearing the MFS.

	Region	ICC (3,1)	SEM (kPa)	Repeatability rating	CV [range]
Mean pressure (kPa)	Whole foot	0.94	6	Excellent	0.03 [0.01–0.07]
	Rearfoot	0.84	5	Good	0.04 [0.01–0.11]
	Midfoot	0.93	2	Excellent	0.05 [0.02–0.10]
	Forefoot	0.93	6	Excellent	0.04 [0.01–0.11]
Peak pressure (kPa)	Whole foot	0.97	12	Excellent	0.03 [0.01–0.08]
	Rearfoot	0.97	8	Excellent	0.03 [0.01–0.06]
	Midfoot	0.96	4	Excellent	0.05 [0.03–0.11]
	Forefoot	0.97	13	Excellent	0.04 [0.01–0.08]

**TABLE 3:** Distribution and repeatability of lower limb joint Range of Motion (ROM) in the sagittal plane across three walking sessions wearing the MFS.

	Session number	ROM (deg, median [25%, 75%])	ICC (3,1)	SEM (deg)	CV [range]
Hip	1	41 [40, 46]	0.47	3	0.06 [0.02–0.10]
	2	43 [40, 44]			
	3	45 [41, 46]			
Knee	1	60 [54, 65]	0.57	4	0.05 [0.01–0.11]
	2	58 [57, 66]			
	3	60 [58, 67]			
Ankle	1	21 [17, 26]	0.81	2	0.08 [0.04–0.10]
	2	21 [19, 24]			
	3	23 [19, 25]			

Analysis of the stability of the fixation between the MFS upper and midsole revealed very small relative displacements in gait (Supporting Information S1: Appendix B). The RMSE of the displacement between markers in the rearfoot region of the MFS was slightly larger than that in the control shoe (1–1.5 mm vs. 0.5–1 mm), whereas no major differences were observed for the markers on the forefoot.

Participants rated the MFS slightly higher for overall comfort than the control shoe (Table 4). No significant differences were found regarding scores for stability or comfort at push‐off between the two conditions.

**TABLE 4:** Outcome of the VAS‐based perceived overall comfort, stability, and push‐off comfort in the MFS and the control shoe conditions.

	MFS score median (IQR [25%, 75%])	Control score median (IQR [25%, 75%])	Wilcoxon *p*‐value
Overall comfort	77.4 [73.2, 87.1]	75.3 [66.8, 82.1]	0.023*
Stability	75.3 [70.8, 86.1]	73.7 [64.7, 77.9]	0.065
Comfort at push‐off	78.9 [67.9, 86.8]	76.8 [67.4, 81.1]	0.797

* indicate statistically significant differences between conditions (*α* = 0.05).

## 4 Discussion

The present study aimed to assess the repeatability of a novel tool designed to facilitate the analysis of the effects of isolated midsole modifications on lower‐limb biomechanical parameters.

The present MFS was developed through a trial‐and‐error process by testing several fixation solutions. The aim was to create a tool that allows quick and repeatable midsole changes without affecting plantar pressure measurements relative to standard footwear. Earlier fixation designs (e.g., using 3D‐printed custom pins or screws to attach the midsole to the upper) presented some limitations, including the time and effort required to change the midsoles, and the durability or strength of the fixation. Alternative attachment methods (e.g., dovetail joints) were also considered; however, the discontinuity in the sole could affect plantar pressure measurements. Fasteners [18] and screws [14] to secure changeable midsole components, or cable ties [19] and cuts in midsoles [4] have also been reported to modify the mechanical properties of the midsoles without changing the upper. However, none of these methods were properly validated, thus making it difficult to determine whether the observed outcomes are solely due to the intended modifications or could be affected by the fixation method. Additionally, some of these designs are limited in the number of interventions that can be tested. For these reasons, a footwear setup using micro hook‐and‐loop fasteners was ultimately identified as the best solution.

According to the study's findings, the MFS enables a quick, strong, and reliable attachment of the midsole to the upper. Furthermore, the current setup does not require pressure insoles and IMUs to be removed or displaced while testing different midsole interventions, thereby potentially enhancing measurement repeatability and reducing testing time. Although the former allows to improve the statistical power when comparing several interventions, the latter is particularly critical in clinical settings where the allocated time for a patient's visit is limited.

Despite the flexible materials used for the uppers, the RMSE of the displacements between upper and midsole components in the MFS during gait were in the range 0.5–1.5 mm and did not show major differences compared with those in the control shoe. None of the participants experienced midsole detachment during walking and in any of the dynamic tasks performed during the familiarization procedure in laboratory conditions.

No differences in joint motion during gait were observed between the two shoe conditions (Figure 2). Additionally, the plantar pressure distributions and magnitudes while wearing the MFS closely resembled those seen in the control shoe, except for the peak pressure at the rearfoot. This small variation in this region may be explained by the fixation layer between the midsole and the upper, which appeared to slightly decrease the cushioning effect during the heel‐strike phase. However, although the participants rated the MFS to be slightly more comfortable than the control shoe, there was no statistically significant difference in participants' perceptions of walking stability and comfort at push‐off. These results, although preliminary, show that the Velcro fixation layer does not significantly affect the biomechanical outcomes of the MFS with respect to those of a standard off‐the‐shelf shoe featuring the same midsole permanently attached to the upper. In fact, the primary purpose of the MFS was not to replicate the biomechanical behavior of the control shoe using a modular design, but to serve as a consistent platform for evaluating the biomechanical effects of different midsole interventions.

The inter‐session variability of plantar pressure parameters observed for the MFS is comparable to that reported for factory‐made and conventional footwear [20, 21, 22, 23]. Across three sessions, both mean and peak pressure demonstrated good‐to‐excellent repeatability (ICC [2, 17] = 0.84–0.97). Thus, the MFS does not seem to introduce abnormal or excessive variability in pressure parameters across sessions. This indicates that the small differences observed are likely due to normal biological and gait variations [20, 21]. Notably, the MFS appears to mitigate the regional variability often observed in studies reporting plantar pressure. Although the midfoot is frequently identified as a region of lower repeatability in barefoot walking [22], and in clinical shoe measurements, where it often displays the highest variability [23], the MFS showed excellent repeatability in this region (ICC [2, 17] = 0.93, 0.96 for mean and peak pressures, respectively).

Although the ankle ROM showed good inter‐session repeatability (ICC [2, 17] = 0.81), the hip and knee ROM measurements demonstrated poor‐to‐moderate repeatability (ICCs [2, 17] = 0.47 and 0.57, respectively). However, absolute repeatability was high across all three walking sessions, showing an SEM ≤ 4 deg and a CV ≤ 8% across all joints. This indicates that the MFS provides a repeatable gait pattern across multiple sessions, and the protocol is resilient to inter‐session variability arising from IMU sensor placement.

The results of this study should be interpreted considering some limitations. In terms of sample size, only 10 participants were recruited, which may reduce the robustness of the results and increase the likelihood of false trends. As for the outcome measures, only biomechanical parameters during walking under laboratory conditions were analyzed. However, bench testing to characterize the mechanical properties of the MFS and midsoles will be performed in future endeavors. With respect to the MFS design, whereas the micro‐hook‐and‐loop fastener layer showed high attachment strength during gait, alternative midsole/upper fixation methods could be devised to provide a stronger connection for highly dynamic tasks such as running and other applications that require greater stability. Moreover, only one upper condition was tested to evaluate the efficacy of the MFS. Testing and validating the connection strength across various upper designs and over longer durations could further broaden the applicability of this tool in footwear research. Finally, although the influence of the Velcro fixation layer on plantar pressure was limited, the observed differences indicate that the in‐shoe pressure data obtained with the MFS may not fully represent those of footwear assembled using conventional industrial manufacturing methods.

## 5 Conclusions

This study reports on the development and preliminary assessment of a new modular tool for testing the isolated effects of midsole designs on biomechanical parameters and perceived comfort. The MFS has shown high repeatability for in‐shoe pressure and kinematic measurements across sessions and could largely replicate the biomechanical performance of a standard off‐the‐shelf shoe featuring the same midsole. Although the MFS requires further validation using different midsole shapes with varying mechanical properties and across more demanding scenarios and motor tasks, the findings of the present study suggest that the MFS used in this study has the potential to be a reliable tool for evaluating the effects of midsole features on relevant biomechanical parameters. This modular approach may enhance data‐driven footwear design by providing a robust, simple‐to‐use, and replicable methodological framework to test the biomechanical effects of midsole designs and materials.

## Author Contributions

**Hadi Sarlak:** conceptualization, data curation, formal analysis, methodology, validation, visualization, writing – original draft, writing – review and editing. **Kamran Shakir:** conceptualization, data curation, formal analysis, methodology, validation, visualization, writing – original draft, writing – review and editing. **Giulia Rogati:** methodology, supervision, validation, writing – review and editing. **Giorgia Sartorato:** methodology, writing – review and editing. **Alberto Leardini:** project administration, resources, supervision, validation, writing – review and editing. **Lisa Berti:** project administration, supervision, funding acquisition, writing – review and editing. **Paolo Caravaggi:** conceptualization, formal analysis, methodology, supervision, validation, funding acquisition, writing – original draft, writing – review and editing.

## Ethics Statement

This study received approval from the local ethical committee (AVEC #647/2025/Sper/IOR).

## Consent

All participants were informed about the data collection process and provided informed consent to participate.

## Conflicts of Interest

G.S. is a collaborator with Podartis SRL, Italy. All other authors declare no financial or nonfinancial competing interests.

## Supporting information


Supporting Information S1

## Data Availability

Further detailed information on the procedure to make the MFS and the CAD files of the 3D‐printed cunei will be made available upon reasonable request to the corresponding author.
