# Supplementary material for: Development of a Novel Modular Footwear Setup for Testing the Isolated Biomechanical Effects of Footwear Features
Source: J Foot Ankle Res. 2026 Aug 21;19(3):e70203. doi: 10.1002/jfa2.70203 (PMC13496383; doi:10.1002/jfa2.70203)
Supplement: Supplementary file 1 [file JFA2-19-e70203-s001.docx]

**Development of a novel Modular Footwear Setup for testing the isolated biomechanical effects of footwear features**

Running title: Validation of a Modular Footwear Setup

**Hadi Sarlak^1,2,†^, Kamran Shakir^1,2,*,†^, Giulia Rogati^2^, Giorgia Sartorato^3^, Alberto Leardini^2^, Lisa Berti^1,4^, and Paolo Caravaggi^2^**

^1^ Department of Biomedical and Neuromotor Sciences, Alma Mater Studiorum - Università di Bologna, 40136 Bologna, Italy; [kamran.shakir2@unibo.it](mailto:kamran.shakir2@unibo.it) (K.S.); [hadi.sarlak2@unibo.it](mailto:hadi.sarlak2@unibo.it) (H.S.); [lisa.berti@unibo.it](mailto:lisa.berti@unibo.it) (L.B.)

^2^ Movement Analysis Laboratory and Functional Evaluation of Prostheses, IRCCS Istituto Ortopedico Rizzoli,

Via di Barbiano 1/10, 40136 Bologna, Italy; [giulia.rogati@ior.it](mailto:giulia.rogati@ior.it) (G.R.); [leardini@ior.it](mailto:leardini@ior.it) (A.L.) [paolo.caravaggi@ior.it](mailto:paolo.caravaggi@ior.it) (P.C.)

^3^ Independent Researcher; sartorato@tbassociati.it (G.S.)

^4^ Physical Medicine and Rehabilitation Unit, IRCCS Istituto Ortopedico Rizzoli, 40136 Bologna, Italy

^†^ These authors contributed equally to this work.

* Corresponding author: Department of Biomedical and Neuromotor Sciences ALMA MATER STUDIORUM - Università di Bologna - Via Zamboni, 33 - 40126 Bologna, Italy; P.No: +39-051-6366571, E-mail address: kamran.shakir2@unibo.it (K.S.)

**Appendices**

# Appendix A: SPM analysis of the temporal profiles of the hip, knee, and ankle joint rotations in the sagittal and frontal planes

# Appendix B: RMSE of the displacement of 4 marker pairs while walking, MFS vs Control
